# Elderly men and women with similar areal bone density show significant differences in bone microstructure and fracture pattern

**DOI:** 10.20945/2359-4292-2026-0126

**Published:** 2026-10-11

**Authors:** Eduardo Medeiros Ferreira da Gama, Leonardo Vieira Neto, Francisco de Paula Paranhos-Neto, Laura Maria Carvalho Mendonça, Luiza Silva Machado de Andrade, Miguel Madeira, Guilherme Cardenaz de Souza, João Antonio Matheus Guimarães, Maria Eugênia Leite Duarte, Maria Lucia Fleiuss Farias

**Affiliations:** 1 Divisão de Endocrinologia, Hospital Universitário Clementino Fraga Filho, Universidade Federal do Rio de Janeiro, Rio de Janeiro, RJ, Brasil; 2 Divisão de Reumatologia, Hospital Universitário Clementino Fraga Filho, Universidade Federal do Rio de Janeiro, Rio de Janeiro, RJ, Brasil; 3 Bone D Consultoria e Treinamento, São Paulo, SR Brasil; 4 Instituto Nacional de Traumatologia e Ortopedia, Divisão de Pesquisa, Rio de Janeiro, RJ, Brasil

**Keywords:** DXA, osteoporosis, TBS, fractures

## Abstract

**Objective:**

To compare bone microarchitecture and fracture patterns between elderly men and women with similar spine and hip bone mineral densities (BMDs).

**Subjects and methods:**

Twenty-eight men and 84 age-BMI-BMD-matched women aged 65 years and older with at least one major osteoporotic fracture underwent laboratory tests, dual-energy X-ray absorptiometry (DXA), high-resolution peripheral quantitative computed tomography (HR-pQCT), and trabecular bone score (TBS).

**Results:**

There were no sex differences in biochemistry, BMD or lowest T-scores. TBS values were low in 67% of the cohort and significantly correlated with HR-pQCT parameters. At HR-pQCT evaluation, men had greater bones (total area and cortical perimeter at distal radius and tibia) and better trabecular vBMD and microstructure at distal radius, but similar values for cortical vBMD and cortical thickness. When compared with Brazilian age-sex-reference HR-pQCT data from healthy individuals, all parameters tested were numerically lower in the study group. These differences seemed more pronounced in men, particularly for cortical thickness at the distal tibia, where relative deficits reached -46.9% in men and -35.4% in women. There was no sex difference in vertebral fractures, but wrist and shoulder fractures were more common in women (p = 0.006), whereas hip fractures were more common in men (p = 0.03).

**Conclusion:**

To achieve similar DXA values, men appear to have greater relative bone deficits than women when compared to age-sex-matched healthy Brazilian population. This study highlights the importance of HR-pQCT to understand sex-specific differences in fracture pattern in the study population.

## INTRODUCTION

Osteoporosis, a systemic skeletal disease characterized by low bone mass and microarchitectural deterioration, significantly increases the risk of fragility fractures in aging individuals of both sexes. Often referred to as a “silent disease“, osteoporosis typically presents no overt symptoms until fragility fractures occur. Importantly, low-impact fractures are frequently unrecognized as indicators of bone fragility, and many patients are not prescribed antiosteoporosis medications. Bone densitometry is a useful method for identifying people at risk of osteoporotic fracture; it involves comparing a patient's bone mineral density (BMD) with a young adult reference range, and the difference is defined as the T-score. Postmenopausal women are frequently asked to undergo BMD measurement, but BMD in elderly men is rarely investigated. Furthermore, fractures frequently arise in individuals who do not meet the established osteoporosis thresholds defined by areal BMD (^1^, ^2^). In fact, osteoporosis remains underdiagnosed and undertreated, particularly in men (^3^, ^4^).

Over the past decades, evolving guidelines from the International Society for Clinical Densitometry (ISCD) have addressed these risks, introducing pivotal updates to diagnostic standards (^5^, ^6^, ^7^, ^8^, ^9^). Prior to 2013, T-scores in men were calculated using male-specific normative reference data, which implies that men were diagnosed as osteoporosis (T score <-2.5 SD) with higher bone density than women. The 2013 ISCD Position Statement recommended using a unified reference population (young adult Caucasian women from NHANES III) to align with WHO criteria, as fragility fractures are expected to occur at similar BMD thresholds in both genders. However, considering differences in bone acquisition during adolescence and early adulthood, and higher peak bone mass in men, comparable dual-energy X-ray absorptiometry (DXA) data suggests a greater bone deficit in males since youth to attain same osteoporosis threshold as women (^10^, ^11^).

Advanced imaging modalities, such as trabecular bone score (TBS) measurement and high-resolution peripheral quantitative computed tomography (HR-pQCT), now provide more detailed evaluations of bone quality and microarchitecture beyond conventional DXA and reveal sex-specific differences in bone fragility, emphasizing a need for tailored approaches to osteoporosis management (^12^, ^13^, ^14^, ^15^).

The aim of this study was to compare bone geometry, volumetric density, and microarchitecture between men and women with similar areal BMDs using HR-pQCT and TBS. Additionally, this study evaluated the prevalence of vertebral and nonvertebral fractures.

## SUBJECTS AND METHODS

### Study population

This cross-sectional study included patients from the Osteoporosis Prevention Study (OSTEOPREV), which was held at the National Institute of Traumatology and Orthopedics and Federal University of Rio de Janeiro in Rio de Janeiro, Brazil. The study was performed under approval by the Institute Review Board (IRB no. 0005.0.305.000-09), and all participants provided written informed consent before study entry.

The aim of the OSTEOPREV study was to evaluate patients > 60 years who sustained a low-impact (fragility) fracture at > 50 years of age, especially those considered as major osteoporotic fractures: wrist, shoulder, ribs, hip, ankle or vertebrae (including nonclinical vertebral fractures detected on radiological examination). Fractures of the skull, facial bones, metacarpals, fingers, and toes were not considered. The exclusion criteria were alcoholism (> 3 drinks per day), malignancies, acquired immunodeficiency syndrome (AIDS), endogenous or exogenous hypercortisolism, thyroid dysfunction, primary hyperparathyroidism, and impaired renal function (glomerular filtration rate below 30 mL/min/1.73 m^2^). Patients who received anti-osteoporosis drugs within the year prior to study enrollment were also excluded. Patients with type 2 diabetes mellitus (T2DM) on controlled dietary therapy and metformin were included in the study.

A total of 750 patients who met the inclusion and exclusion criteria were referred for radiological evaluation. Finally, 32 men and 308 women were selected after confirmation of having prevalent non-vertebral (wrist, shoulder, hip) or vertebral fractures. These 340 patients were further evaluated for body mass index (BM I: weight divided by the squared height), biochemical tests and bone densitometry (dual-energy X-ray absorptiometry, DXA) and bone tomography (high-resolution peripheral quantitative computed tomography, HR-pQCT). Ultimately, 28 men and 277 women completed all the exams.

Across the whole cohort, men were older, had lower BMI and higher DXA values at all central sites, but had more hip fractures than women. To ensure methodological adequacy in sex comparisons, a subset of 84 women was selected considering age >65 years, body mass index lower than 33 kg/m^2^ and BMD values equal or higher than 0.850 g/cm^2^ at the lumbar spine and 0.725 g/cm^2^ or higher at the femoral neck and total hip. This matching process was implemented to minimize the potential confounding effects of these factors on the study outcomes. The selection process, including screening, eligibility, and matching procedures, is summarized in **Figure 1.**

**Figure 1:**
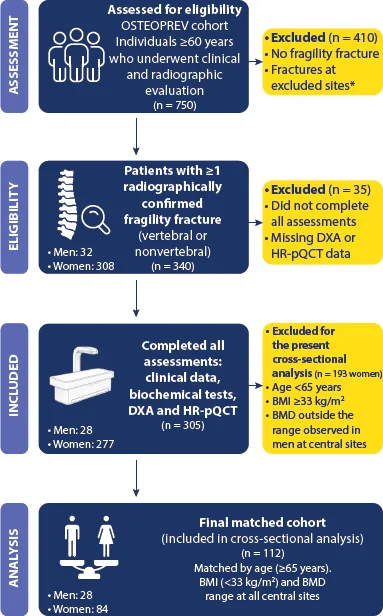
Flowchart of the study participant selection and matching process. The flowchart illustrates the stages of the OSTEOPREV cohort selection, from the initial screening of individuals with suspected f ragi I ity f ractu res to the fi na I matched a na lysis grou p. Assessment: 750 individuals were initially evaluated; eligibility: 340 patients (32 men and 308 women) had radiographically confirmed fragility fractures; included: 305 patients completed the full suite of exams (DXA, HR-pQCT, and TBS); analysis: Afinal matched cohort of 112 individuals (28 men and 84 women) was selected based on age, BMI, and BMD ranges to ensure methodological adequacy for sex-based comparisons.

*Excluded sites: skull, facial bones, metacarpals, fingers, toes.

DXA: dual-energy X-ray absorptiometry: HR-pQCT: high-resolution peripheral quantitative computed tomography; BMI: body mass index; BMD: bone mineral density.

### Radiographic assessment

Frontal and lateral plain radiographs of the thoracic and lumbar spine were obtained to identify and classify vertebral fractures (VFxs) according to the semiquantitative method of Genant (^16^). Vertebral body deformities were graded as 1 (mild: 20%-25%), 2 (moderate: 25%-40%), or 3 (severe: > 40%). Nonvertebral fractures (hip, wrist and shoulder) were confirmed based on clinical history and radiographic documentation, either at fracture occurrence or at the study.

### DXA

Areal BMD (g/cm^2^) of the lumbar spine, femoral neck, and total hip was measured using a GE Lunar Prodigy Advance device (GE Healthcare Madison, Wisconsin, USA) with enCore V16 (SP2) software. Across the entire cohort, T-scores were calculated using the NHANES III database for Caucasian females aged 20-29 years as the reference standard. In accordance with the 2023 ISCD guidelines (^9^), patients were classified as having normal BMD, low BMD (osteopenia), or osteoporosis based on the lowest T-score value (> -1 SD, between < -1 and > -2.5 SD, and < -2.5 SD, respectively).

### TBS

Trabecular bone score (TBS) was calculated using TBS iNsight software (Medlmaps, version 2.1) on the same region of interest as the lumbar spine BMD. For statistical analysis, patients were categorized into two groups: those with normal bone microstructure (TBS ≥ 1.310) and those with abnormal bone microstructure (TBS < 1.310). The latter group included individuals with both partially degraded and degraded bone microstructures, as defined by McCloskey and cols. (2016) (^17^).

### HR-pQCT

Volumetric bone mineral density (vBMD) and microarchitecture were measured using a first-generation HR-pQCT system (XtremeCT I; Scanco Medical AG, Brüttisellen, Switzerland) following the manufacturer's instructions. This method enables the separate assessment of cortical and trabecular compartments at the nondominant distal forearm and distal tibia. Data acquisition and analysis adhered to the recommendations outlined in a recent consensus (^18^). vBMD was measured for the entire bone [Tt.BMD], cortical bone [Ct.BMD], and trabecular bone [Tb.BMD]. The microstructural parameters included cortical thickness [Ct. Th: ratio of cortical bone volume to the outer bone surface], trabecular number [Tb.N], trabecular bone volume fraction [BV/TV: trabecular density/1200 mg hydroxyapatite], trabecular thickness [Tb.Th: (BV/ TV)/Tb.N], trabecular separation [Tb.Sp: (1-BV/TV)/ Tb.N], and trabecular inhomogeneity [Tb.1/N.SD, the standard deviation of 1/Tb.N]. Bone geometry included cortical and trabecular areas [Ct.Ar, Tb.Ar] and the cortical perimeter [Ct.Pm]. The coefficient of variation for HR-pQCT in our institution is 3-5% for microarchitectural parameters and 1-2% for density measurements. CT images with motion artifacts classified as 3 or higher (^18^) were excluded from analysis.

### Biochemistry

Blood samples were collected between 8 AM and 10 AM after an overnight fast. Serum calcium (RV 8.5-10.1 mg/dL), phosphorus (RV 3.0-4.5 mg/dL), albumin (RV 3.5-5.5 g/dL) and creatinine (RV 0.8-1.3 mg/dL) concentrations were measured using standard laboratory methods. The glomerular filtration rate (GFR) was estimated using the Chronic Kidney Disease Epidemiology Collaboration equation (^19^). Serum 25-hydroxyvitamin D (25(OH)D) levels were assayed using electrochemiluminescence (Elecsys kit 2010, Roche, Berlin, Germany; RV 30-100 ng/mL). Intact parathyroid hormone (PTH) levels were measured using a chemiluminescence immunoassay (IMMULITE 2000 kit; Siemens, Munich, Germany; RV 11-65 pg/mL). Bone resorption was assessed by measuring serum levels of the carboxyterminal telopeptide of type I collagen (CTX) using an electrochemiluminescence immunoassay (β-Cross Laps; Roche Diagnostics, Mannheim, Germany). The reference range for the concentration of CTX was 0.035-0.836 ng/mL in men aged 50 years and older, < 0.573 ng/mL in premenopausal women and < 1.00 ng/mL in postmenopausal women.

### Statistics

All the statistical analyses were conducted using SPSS version 27.0 for MacOS X (SPSS, Inc., Chicago, IL, US). Descriptive statistics are presented as follows: categorical variables are expressed as frequencies and percentages, whereas continuous variables are expressed as the means ± standard deviations (SDs) or medians with the first and third quartiles, as appropriate. The normality of the data distribution was assessed using the Kolmogorov-Smirnov test. For between-group comparisons, Student's *t* tests or Mann-Whitney U tests were employed for continuous variables, depending on the normality of the data distribution. To assess the magnitude of differences of HR-pQCT variables between genders, effect sizes were calculated using Cohen's, where values of 0.2, 0.5, and 0.8 are considered small, medium, and large effects, respectively. Categorical variables were compared using the chi-square test or Fisher's exact test, as appropriate. Spearman's rank correlation coefficient was used to assess correlations between continuous variables. Multivariate analysis considered the entire cohort to test association of TBS and HR-pQCT parameters (total area, cortical perimeter, Tb.vBMD and Tb.N) with wrist-shoulder fractures (n = 37) and with hip fractures (n = 12). Statistical significance was defined as a p value less than 0.05.

## RESULTS

This study included 28 men and 84 women with prevalent low-impact fractures, fulfilling the diagnostic criteria for bone fragility. Baseline characteristics and biochemical parameters were comparable between men and women. Consistent with the initial study objectives, there was no sex difference with respect to the lumbar spine, femoral neck, or total hip BMD; the median values of the lowest BMD T-score; or the proportion of individuals classified as having osteoporosis according to the DXA criteria (35.7% of men and 34.5% of women). These data are shown in **Table 1.**

**Table 1:** Characteristics of male and female patients with similar areal BMD and classification as osteoporosis based on the lowest T-score using NHANES III data of young women for both genders

	**Males n=28**	**Females n=84**	***P***
Anthropometry and biochemistry			
Age (years)	72.61 ±5.52	72.32 ±6.77	0.842
Body Mass Index (kg/m^2^)	26.42 ±4.87	28.08 ±3.73	0.062
Type 2 diabetes mellitus N (%)	5(17.9)	11 (13.1)	0.500
GFR(mL/min/1.73m^2^)	70.75 ±14.53	75.64 ±14.80	0.132
25(0H)D (ng/mL)	31.61 ±19.19	27.58 ±13.39	0.223
PTH (pg/mL)	42.53 ±18.91	45.32 ±18.46	0.492
ALP(pg/mL)	30.54 ±14.30	30.80 ±16.46	0.940
CTX (ng/mL)	0.332 ±0.163	0.306 ±0.173	0.484
DXA			
Lumbar spine BMD(g/cm^2^)	1.086 ±0.258	1.050 ±0.193	0.425
Femoral neck BMD (g/cm^2^)	0.866 ±0.141	0.845 ±0.138	0.489
Total hip BMD (g/cm^2^)	0.912 ±0.144	0.878 ±0.146	0.276
Lowest BMD T-score	-2.15 (-2.60;-0.83)	-2.00 (-2.60;-1.02)	0.870
Osteoporosis based on DXA criteria N (%)	10(35.7)	29 (34.5)	0.910

GFR: glomerular filtration rate estimated by CKD-EPI; 25(0H)D: 25-hydroxyvitamin D; PTH: parathyroid hormone; ALP: alkaline phosphatase; CTX: C-terminal telopeptide of type I collagen. Values expressed as mean (SD), median (IQR) or absolute number (percentage).

## HR-pQCT and TBS

HR-pQCT analysis revealed significant sex-based differences with large to very large effect sizes in bone geometry, density and microarchitecture. At the distal radius, very large effects were observed for total area (d = 2.41), trabecular area (d = 2.24), and cortical perimeter (d = 2.46). Significant microstructural differences were also supported by large effects in BV/TV (d = 1.00) and trabecular BMD (d = 0.74). Men exhibited larger bones, characterized by higher values for total, cortical, and trabecular areas and cortical perimeter at both the radius and tibia (p < 0.001 for all comparisons). At the distal radius, men also had higher values for trabecular vBMD, BV/TV, trabecular number, and lower trabecular separation (p < 0.02 for all comparisons). On the other hand, there was no sex-based differences in cortical vBMD and thickness, suggesting more intense modifications in cortical bone in those elderly men than in elderly women.

As the cross-sectional design does not allow evaluation of longitudinal skeletal changes, our cohort was compared with age-sex-specific normative HR-pQCT data obtained from healthy Brazilian population, without fractures as established by Alvarenga and cols. (^20^, ^21^). Median values of all HR-pQCT parameters tested were numerically lower in the study group. These differences seemed more pronounced in men, particularly for cortical thickness at the distal tibia, where relative deficits reached -46.9% in men and -35.4% in women. These comparisons are shown in **Table 2** and **Figure 2.**

**Table 2:** Relative differences of HR-pQCT parameters between the Osteoprev fracture cohort and age-and sex-mached Brazilian normative reference populations reported by Alvarenga and cols. (^20^, ^21^)

	**Control group**	**Osteoprev 60-69 yrs.**	**60-69**	**Control group ≥70 yrs.**	**Osteoprev ≥70 yrs.**	**A% ≥70**	**Relative difference (%) since 60 yrs.**
Men							
Tb.BMD radius (HA/cm^3^)	180.3(148.2-195.6)	149.4(133.7-206.5)	-17.1	181.5(154.2-216.3)	159.6(126.8-172.8)	-12	-29.1
Tb.BMD tibia (HA/cm^3^)	158.3(149.2-186.7)	143.7(113.3-168.7)	**-5.6**	169(140.8-178.3)	140.7(116.3-162.7)	-16.7	-22.3
Tb.N radius (mm^1^)	2.08(1.82-2.30)	2.08(1.68-2.22)	**0**	2.14(1.90-2.35)	1.86(1.70-2.02)	-13.1	-13.1
Tb.N tibia (mm^1^)	1.76(1.62-2.11)	1.65(1.47-1.98)	**-6.2**	1.91(1.60-2.14)	1.65(1.33-1.77)	-13.6	-19.8
Ct.BMD radius (HA/cm^3^)	941(916.5-979.7)	850.2 (806.7-874.3)	**-9.6**	931.5(899.6-968.3)	813.9(759.0-836.5)	-12.6	-22.2
Ct.BMD tibia (HA/cm^3^)	913(888.7-952.2)	843.5(801.9-853.3)	**-7.6**	890.9 (837.8-932.8)	835.9(762.5-875.2)	-6.2	-13.8
Ct.Th radius (mm)	0.864(0.761-0.945)	0.700(0.610-0.790)	**-19**	0.899(0.756-1.045)	0.650 (0.508-0.693)	-27.7	-46.7
Ct.Th tibia (mm)	1.253(1.140-1.397)	0.955(0.888-1.190)	-23.8	1.321 (1.100-1.425)	1.015(0.795-1.218)	-23.1	-46.9
Women							
Tb.BMD radius (HA/cm^3^)	142(127-163)	138.50(104.95-157.65)	**-2.5**	137(115-158)	126.10(94.9-148.6)	-8.0	-10.5
Tb.BMD tibia (HA/cm^3^)	137(117-158)	133.00(109.80-168.50)	**-2.9**	136(119-170)	128.10(103.9-153.5)	-5.8	**-8.7**
Tb.N radius (mm^1^)	1.88(1.65-2.05)	1.82(1.63-2.01)	**-3.2**	1.85(1.67-2.02)	1.62(1.40-1.90)	-12.4	-15.6
Tb.N tibia (mm-1)	1.60(1.37-1.78)	1.53(1.28-1.83)	**-4.4**	1.57(1.28-1.87)	1.54(1.33-1.72)	-1.9	**-6.3**
Ct.BMD radius (HA/cm^3^)	968(932-1016)	840.20(801.85-870.95)	-13.2	925 (868-972)	855.40(811.40-887.00)	-7.5	-20.7
Ct.BMD tibia (HA/cm^3^)	903 (873-934)	819.70(778.80-848.60)	**-9.2**	830 (806-882)	811.30(780.15-875.85)	-2.3	-11.5
Ct.Th radius (mm)	0.830(0.720-0.910)	0.650(0.555-0.730)	-21.7	0.780 (0.630-0.870)	0.640 (0.530-0.780)	-17.9	-39.6
Ct.Th tibia (mm)	1.140(0.910-1.310)	0.960(0.810-1.070)	-15.8	1.120(0.930-1.310)	0.900(0.735-1.180)	-19.6	-35.4

Values are presented as median (IQR). Δ% refers to the percentage differences between Osteoprev and control groups described in references 20 and 21.

**Figure 2:**
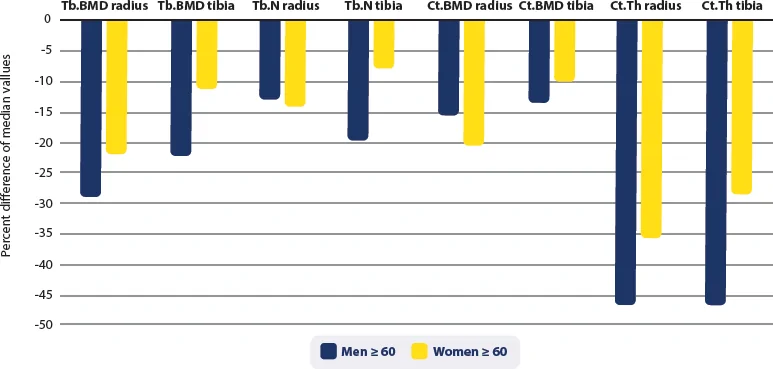
Relative deficits of HR-pQCT parameters obtained in the Osteoprev study group as compared to Brazilian healthy men and women, taken as control group (^20^, ^21^).

Baseline (0) represents median values obtained in the reference age-sex-healthy population. Bars indicate percentage differences for men and women with fragility fractures relative to reference values.

The absolute values of the trabecular bone score (TBS), a surrogate marker of bone microarchitecture, were below normal limits (1.310) in *67%* of the cohort (53.6% of males and 71.4% of females; p = 0.08 for sex comparison). These data are presented in **Table 3.** Furthermore, the TBS was significantly correlated with the lowest BMD T-score (r = 0.66 *p* = 0.04), cortical vBMD at the distal radius (r = 0.21 *p* = 0.02) and distal tibia (r = 0.29 *p* = 0.002) and cortical thickness at the tibia (r = 0.30 *p* = 0.002), suggesting an interplay between trabecular and cortical compartments in bone quality assessment.

**Table 3:** Bone assessment by TBS and HR-pQCT in men and women with similar aBMD

	**Males n=28**	**Females n=84**	***P***	**Cohen's *d***
HR-pQCT (radius)				
Total area (mm^2^)	359.88 ±60.56	251.22 ±38.78	**&lt;0.001**	2.41
Cortical area (mm^2^)	54.16 ±15.42	44.64 ±12.41	**0.002**	0.72
Trabecular area (mm^2^)	295.18 ±57.68	199.06 ±36.91	**&lt;0.001**	2.24
Total BMD (HA/cm^3^)	273.98 ±58.29	273.83 ±60.42	0.963	
Cortical BMD (HA/cm^3^)	821.46 ±100.90	842.03 ±73.00	0.264	
Cortical thickness (mm)	0.65±0.18	0.66 ±0.18	0.808	
Cortical perimeter (mm)	83.60 ±8.42	68.02 ±5.48	**&lt;0.001**	2.46
Trabecular BMD (HA/cm^3^)	150.95 ±41.92	119.71 ±42.35	**0.002**	0.74
BV/TV (^1^)	0.13 ±0.03	0.10 ±0.03	**0.002**	1.00
Trabecular number (mm^1^)	1.92 ±0.35	1.64 ±0.43	**0.003**	0.68
Trabecular thickness (mm)	0.06 ±0.01	0.06 ±0.01	0.089	
Trabecular separation (mm)	0.48 ±0.13	0.61 ±0.26	**0.017**	0.55
Trabecular heterogeneity (mm)	0.23 ±0.11	0.33 ±0.26	0.050	
HR-pQCT (tibia)				
Total area (mm^2^)	850.98 ±130.58	655.26 ±104.13	**&lt;0.001**	**1.76**
Cortical area (mm^2^)	116.95 ± 27.10	94.51 ±27.15	**&lt;0.001**	**0.83**
Trabecular area (mm^2^)	720.65 ±125.08	549.53 ±108.33	**&lt;0.001**	**1.52**
Total BMD (HA/cm^3^)	241.03 ±49.82	245.09 ±57.23	0.745	
Cortical BMD (HA/cm^3^)	819.09 ±66.01	820.96 ±75.64	0.910	
Cortical thickness (mm)	1.02 ±0.24	0.93 ±0.29	0.191	
Cortical perimeter (mm)	115.55 ±9.63	102.99 ±16.89	**&lt;0.001**	0.81
Trabecular BMD (HA/cm^3^)	134.28 ±37.89	128.85 ±41.08	0.550	
BV/TV (^1^)	0.11 ±0.03	0.11 ±0.03	0.547	
Trabecular number (mm^1^)	1.63 ±0.36	1.50 ±0.35	0.117	
Trabecular thickness (mm)	0.07 ±0.01	0.07 ±0.01	0.527	
Trabecular separation (mm)	0.58±0.18	0.63 ±0.19	0.202	
Trabecular heterogeneity (mm)	0.30 ±0.14	0.36 ±0.22	0.201	
TBS	1.287 ±0.114	1.260 ± 0.12	0.317	
TBS &lt; 1.310 N(%)	15(53.6)	60(71.4)	0.080	

HR-pQCT: high-resolution peripheral quantitative computed tomography; BMD: bone mineral density. Vaues expressed as mean (SD).

## Fractures

All participants had prevalent fractures, as this was a determinant criterion for the study protocol. A total of 54 fractures were identified in the male group: 46 VFxs classified as grade 1 (n = 30), grade 2 (n = 5), and grade 3 (n = 11) and eight NonVFxs at the hip (n = 6), wrist (n = 1) and shoulder (n = 1). In the female group, 177 fractures were identified: 136 VFx, classified as grade 1 (n = 91, grade 2 (n = 28), and grade 3 (n = 17), and 41 NonVFx, at the hip (n = 6), wrist (n = 24), and shoulder (n = 11). Notably, most patients were unaware of the vertebral fractures, only diagnosed on radiographic examination. There were no significant sex differences in the frequency of vertebral fractures. However, wrist and shoulder fractures were more common in women (*p* = 0.006), whereas hip fractures were more common in men (*p* = 0.03). Multivariate analysis did not determine the influence of TBS or HR-pQCT parameters in prevalent fractures at wrist-shoulder or the hip.

## DISCUSSION

This study highlights significant sex-based differences in bone microarchitecture, volumetric bone density, and fracture pattern among elderly individuals with comparable aBMD, despite the prevailing notion that fractures occur at similar BMD thresholds in both sexes. These findings underscore the critical importance of integrating advanced imaging techniques to comprehensively assess bone fragility and guide the development of personalized management strategies.

The study included a cohort of 28 men and 84 women whose baseline characteristics were comparable. Key demographic, clinical, and laboratory variables, including age, GFR, 25(OHD) level, and PTH level, did not significantly differ between sexes, indicating that the comparisons between groups were robust. Serum levels of C-terminal telopeptide of type I collagen (CTX), a biomarker of bone resorption, were within the expected range in all participants, indicating the absence of recent bone fractures (^22^). These findings support the hypothesis that bone fragility in this cohort is primarily attributable to chronic structural deterioration rather than accelerated bone turnover.

Standardizing the diagnosis of osteoporosis in both sexes by comparing patient BMD with data obtained from young women assumes that fracture risk is based on absolute BMD values. Considering the distinct trajectories of bone acquisition in men and women during adolescence and early adulthood, healthy men develop bigger bones and higher bone density. Therefore, achieving similar DXA values may require greater relative skeletal deficits in men then in women.

Notably, skeletal changes involve biological and hormonal factors, including the growth hormone (GH) and insulin-like growth factor-1 (IGF-1) axes, as well as sex steroids. Testosterone, in particular, contributes to the larger skeletal size and thicker cortices of the adult male skeleton (^23^). Females typically reach peak bone mass earlier, with minimal gains occurring 2-4 years postmenarche, whereas males experience prolonged bone accumulation into their late teens and early twenties, resulting in greater variability and differences in peak bone mass attainment (^24^). Studies using HR-pQCT have confirmed these sex-based differences, demonstrating that compared with women, young men have greater trabecular bone volume because of increased trabecular number and thickness in both the radius and tibia. Moreover, men exhibit a greater total area, cortical area, and cortical thickness, despite greater cortical porosity in the radius and tibia (^15^). These developmental differences establish a foundation for sex-specific skeletal characteristics, with men typically achieving higher muscle mass, bone volume, and bone density than women do. As men and women age, these early skeletal differences translate into distinct patterns of bone changes. Women experience a more rapid rate of bone loss during the first 10 years following menopause due to estrogen deficiency, leading to trabecular thinning and perforation, resulting in reduced connectivity within the trabecular network. In contrast, men experience bone loss at a slower rate and later in life, primarily through trabecular thinning rather than disruption of the trabecular network. Cortical bone modifications typically commences after 65 years of age and progresses at a similar rate in both sexes (^25^). These distinct patterns of bone changes have critical implications for fracture risk: Women face a higher risk of fractures earlier in life, particularly in trabeculae-rich bones such as the vertebrae, wrist, and shoulder, due to the increased metabolic activity of trabecular bone. Conversely, hip fractures, which reflect combined cortical and trabecular involvement, tend to occur later in life (^25^, ^26^).

In our cohort, women exhibited a higher prevalence of wrist and shoulder fractures, whereas hip fractures were more prevalent in men. Although men have larger bone dimensions, geometric parameters have limited predictive value for fragility fractures (^14^). Sex differences may be influenced by several factors, including potential selection bias, such as differences in body composition, physical activity, fall risk and comorbidities, which were not investigated. Another variable is that men may be less likely to seek medical attention or undergo routine bone density screening, leading to a greater proportion of men presenting with more severe fractures, including hip fractures. Alternatively, sex differences in fracture pattern may reflect more pronounced cortical bone deficits in men to reach comparable aBMD at sites with predominantly cortical bone, such as the femoral neck and total hip. This hypothesis is supported by the similarities in cortical BMD and thickness between men and women, as revealed by HR-pQCT analysis. The cross-sectional contrasts with healthy reference values described by Alvarenga and cols, suggested that individuals with fragility fractures exhibited more severe trabecular and cortical microarchitectural deterioration than would be expected from healthy aging, particularly among men.

We emphasize that the aim of our study was to evaluate differences in bone microstructure between men and age-BMD matched women, not to identify HR-pQCT parameters associated with fracture risk. In fact, other publications addressed the relationship between HR-pQCT and fractures, the majority based on elderly women. Vilayphiou N and cols. (^27^) evaluated elderly men from the Structure of the Aging Men's Bones (STRAMBO) cohort and reported that areal and vBMD, cortical thickness and trabecular number, separation, and distribution were significantly worse in those 185 men with prevalent fragility fractures than in controls, with differences ranging from -6% to 15%. µFE-derived stiffness and failure load were 8% to 9% lower in men with fractures. The systematic review and meta-analysis of 25 studies published by Cheung and cols. (^14^) identified stiffness, cortical vBMD, and trabecular thickness as the outstanding parameters for fracture prediction. For this purpose, distal radius was shown to be a more preferable measurement site than distal tibia. It is worth noting that 16 studies included women only, six studies men only and three studies both genders, but eventual differences in HRpQCT parameters between men and women with fragility fractures were not considered.

We acknowledge that HR-pQCT remains primarily a research tool due to its high cost, limited availability, and lack of widespread integration into clinical practice. TBS is a more accessible method and identified abnormal bone microstructure in approximately 2/3 of the whole cohort, despite the lack of significant sex differences. The significant correlations between the TBS and HR-pQCT parameters at cortical bone reinforced the ability of the TBS to complement DXA in the identification of bone fragility, which is consistent with our previous observation (^28^, ^29^, ^30^, ^31^).

Our findings are consistent with the literature, which has demonstrated that most vertebral fractures are only diagnosed by active radiological investigation, and that a significant proportion of low-impact fractures occur in individuals who do not meet the diagnostic criteria for osteoporosis based on BMD measurements. Additionally, we observed a greater frequency of hip fractures in men, suggesting important microstructural changes in men with similar BMD T-score threshold defined for women.

The importance of actively screening and diagnosing osteoporosis in men was emphasized in the latest guidelines from the International Society for Clinical Densitometry (ISCD) (^9^) and the International Osteoporosis Foundation (IOF). These organizations recommend targeted assessments for men at higher risk of osteoporosis, particularly those with risk factors such as advanced age, low body weight, or a history of fractures. However, the United States Preventive Services Task Force (USPSTF) currently states that insufficient evidence exists to support routine osteoporosis screening in men, highlighting a gap in research and a need for stronger clinical data to guide recommendations (^32^). This lack of consensus underscores the challenges in addressing osteoporosis in men and may partially explain the observed delays in diagnosis and undertreatment, which ultimately contribute to a higher frequency of severe outcomes, such as hip fractures, in this population; this is a concerning trend, considering the projected increase in the elderly population. The 2022 Brazilian Institute of Geography and Statistics (IBGE) Census reported a 57.4% increase in the population aged 65 years and older, which now comprises 10.9% of the Brazilian population. Projections indicate that by 2070, 37.8% of the population will be over 60 years of age (^33^, ^34^). Postmenopausal women are generally more aware of their osteoporosis risk than men are; men's risks are often underinvestigated using imaging methods. The BRAZOS Study, published in 2009, reported a prevalence of fragility fractures of 15.1% in women and 12.8% in men aged 40 years and older (^35^). Furthermore, the BRAVOS study, conducted between 2010 and 2012, identified 1025 hip fractures (310 in men and 715 in women) in a merged population of individuals aged 50 years and older from three representative geographic areas in Brazil (^36^). These data highlight the significant burden of fragility fractures in the Brazilian population and emphasize the urgent need for improved osteoporosis screening and management strategies, particularly among men. For both genders, a simple initial assessment can include spine radiography and FRAX evaluation (^37^, ^38^). Whenever possible, DXA and trabecular bone score (TBS) assessments should be incorporated into the diagnostic workup.

Our study has several limitations. The cross-sectional design limits the identification of sex-differences in longitudinal bone changes, as well as the establishment of causal relationships between changes in bone microarchitecture and fracture patterns. Falls can influence the type and severity of fractures, but data on falls were not uniformly available for all participants and could not be considered in the present analysis. However, it is worth noting that most vertebral fractures were detected only on X-ray evaluation and that these morphometric, nonclinical vertebral fractures are unrelated to falls. The relatively small size of the male cohort reflects differences in spontaneous demand for osteoporosis treatment between the sexes. We recognize that this may have limited the comparison of fractures subgroups. On the other hand, the aim of this study was not to define bone alterations associated with fractures but to evaluate if there were differences in bone microstructure between men and age-BMD matched women. The very large effect sizes found in comparisons of geometry and trabecular parameters between men and women (all *d >* 0.70) suggest that these sex-specific differences are biologically robust and unlikely to be due to chance.

Despite these limitations, this study provides important insights into sex-based differences in bone quality and fracture patterns, supporting a more comprehensive approach to bone assessment beyond conventional DXA. Future longitudinal studies with larger sample sizes are warranted to further investigate the long-term impact of sex-specific bone microarchitectural changes on fracture risk.

In conclusion, this study provides key insights into sex-specific disparities in bone microarchitecture, vBMD, and fracture patterns observed among elderly individuals with comparable aBMDs. The integration of advanced imaging techniques, such as TBS and HR-pQCT, can provide valuable information and aid in the development of more precise and personalized strategies for fracture prevention in both men and women. While these findings suggest that men and women may follow distinct skeletal aging trajectories, further studies are needed to determine whether sex-specific diagnostic or treatment approaches are justified.

## Data Availability

datasets related to this article will be available upon request to the corresponding author.
